# ESR1 Regulates the Obesity- and Metabolism-Differential Gene MMAA to Inhibit the Occurrence and Development of Hepatocellular Carcinoma

**DOI:** 10.3389/fonc.2022.899969

**Published:** 2022-06-20

**Authors:** Yiyin Zhang, Jiaxi Cheng, Cheng Zhong, Qiming Xia, Yirun Li, Peng Chen, Xiaoxiao Fan, Qijiang Mao, Hui Lin, Defei Hong

**Affiliations:** ^1^ Department of General Surgery, Sir Run Run Shaw Hospital, School of Medicine, Zhejiang University, Hangzhou, China; ^2^ State Key Laboratory of Modern Optical Instrumentations, Centre for Optical and Electromagnetic Research, College of Optical Science and Engineering, International Research Center for Advanced Photonics, Zhejiang University, Hangzhou, China; ^3^ Zhejiang Engineering Research Center of Cognitive Healthcare, Sir Run Run Shaw Hospital, School of Medicine, Zhejiang University, Hangzhou, China

**Keywords:** obesity, hepatocellular carcinoma, MMAA, ESR1, redox balance

## Abstract

Obesity is often regarded as a factor that promotes tumorigenesis, but the role of obesity in promoting hepatocellular carcinoma (HCC) is still controversial. We compared the trend change of 14 obesity-related genes in the formation and development of HCC in normal, adjacent, and HCC tissues. Mendelian randomization (MR) analysis was used to verify the relationship between obesity and HCC occurrence. Metabolism of cobalamin-associated A (MMAA) was discovered as an obesity- and metabolism-differential gene, and its function in HCC was tested *in vitro* and *in vivo*. Finally, we explored how obese female patients with an originally high expression of female estrogen receptor (ESR1) directly upregulated MMAA to interfere with the progression of HCC. Fourteen obesity-related genes were downregulated in adjacent and tumoral tissues compared with normal liver tissues, which indicated that obesity may be inversely related to the occurrence of HCC and was consistent with the results of MR analysis. We also discovered that MMAA is a metabolic gene closely related to the occurrence and development of HCC by mining the TCGA database, and it functioned an anti-tumor-promoting role in HCC by damaging the mitochondrial function and preserving the redox balance. We further verified that obese females with a high expression of ESR1 can regulate MMAA to protect HCC from progression. This study elucidates that obesity might be a protective factor for female HCC patients, as they originally highly expressed ESR1, which could upregulate MMAA to suppress tumor growth and participate in metabolic reprogramming.

## Introduction

Hepatocellular carcinoma (HCC) constitutes the majority of liver cancer cases and ranks 7th among newly emerging malignant tumors, causing a fatality rate of 8.3% (1). There is a sex preference in the incidence and mortality of HCC, and HCC favors male over female individuals, with an incidence ratio of 7:1–10:1 (2). According to the latest global cancer statistics, the incidence rate of HCC is 6.3% in male and 3.0% in female patients, with mortality rates of 10.5 and 5.7%, respectively (1). Consistently, hepatocarcinogenesis in an animal experiment also supports that male patients are more susceptible to HCC regardless of the etiology (chronic viral infection of hepatitis B or C, smoking, and alcohol abuse) (3). This suggests that the occurrence of HCC may be related to the genetic information carried by different sexes, but the underlying mechanism remains unknown.

Obesity is defined by the World Health Organization as a body mass index (BMI) higher than 30.0 kg/m², and BMI between 25 and 29.9 kg/m² is defined as overweight. However, for Asians and Westerners of the same weight, Asians have a higher body fat content than Westerners but less muscle content. It is therefore recommended that Asians whose BMI exceeds 25.0 kg/m², a relatively safe reference value, be considered obese (4). Obesity, as a factor that promotes tumorigenesis, is second only to smoking (5). Additionally, obesity is commonly believed to potently enhance the risk for tumor progression and recurrence, including breast cancer, ovarian cancer, pancreatic cancer, gastric cancer, and meningioma (6). However, obesity has a counterintuitive association with prognosis, especially in patients treated with immunotherapy. Among 330 patients with advanced chromocytoma treated with PD-1 inhibitors, the average life expectancy of obese patients was 13 months longer than that of patients of normal weight (7). The expression level of PD-L1 increases with body weight regardless of species. Serotonin increases with the gradual growth of adipose tissue until it is similar to cancer cells, and it participates in the regulation of the function of T cells and is positively correlated with the expression of PD-1 checkpoint protein (8). Moreover, adipose tissue produces estrogen, causing the estrogen levels to rise (9); female estrogen receptor (ESR1), which encodes estrogen receptor, is highly expressed in female patients compared with male patients (10). Therefore, it is unclear whether adipose tissue genes could be activated by ESR1 to cause prognostic differences in female HCC patients.

When the adaptive lipid storing function of adipose tissue fails, obesity becomes a metabolic disease that affects the inner membrane functions, submitochondrial localization, and ultrastructural morphology of the mitochondria (11). Obesity could lead to mitochondrial malfunction, manifesting as decreased synthesis and activity of mitochondrial substances, excessive production of reactive oxygen species, and increased autophagy, which can, in turn, affect the function of adipose tissue and result in the whitening of brown fat (12). CB-839 exerts an anticancer effect by reducing the mitochondrial membrane potential and reducing the number of mitochondria, which could significantly inhibit tumor metabolism (13). Recent studies have demonstrated that sex differences also occur in the efficacy of cancer treatment (14). Previous studies have confirmed that, compared with male mitochondria, female mitochondria have a stronger biosynthesis and a stronger antioxidant defense ability, reducing the production of cellular reactive oxygen species (ROS) and thus postponing tumor progression (9). This reminds us not to neglect the association between gender specificity, mitochondria, and cancer. Moreover, Ke *et al.* found that the upregulation of 14 genes leads to obesity; thus, the relationship between the 14 genes and the potential anti/oncogenic genes in HCC should be further explored (15). Metabolism of cobalamin-associated A (MMAA) is the key gene related to mitochondrial function, the lack of which would lead to the development of methyl acryluria eventually progressing to hepatoblastic carcinoma due to the impaired tricarboxylic acid cycles and accumulation of oxidative stress as well as toxic metabolites (16). There is currently no research on the correlation between MMAA and HCC; considering that mitochondrial function changes due to the defect of MMAA, it may have a potential correlation with the occurrence and progression of HCC.

Although many studies have shown that obesity promotes the onset of tumors, they are all based on traditional observational epidemiology methods, which often ignore confounding bias. Especially in HCC, few studies have verified the correlation between obesity and oncogenesis. Mendelian randomization (MR) is a tool for variable analysis that uses genetic variation (such as single-nucleotide polymorphisms, SNPs) as the target to be studied. The variables of the exposure factors can effectively overcome these limitations and clarify the causality correlation between exposure factors and the cause of the tumor (17). In this study, we found that obesity is a protective factor in female HCC through MR analysis, and the obesity differential gene MMAA was selected *via* bioinformatic analysis. We investigated the function of MMAA in HCC and found that the upregulation of MMAA in tumor tissues predicts a longer survival for HCC patients. MMAA suppresses the proliferation and migration of HCC cells and regulates the redox balance, thereby highlighting MMAA as a critical target for the treatment of HCC.

## Results

### Obesity Is a Protective Factor for HCC Occurrence in Female Patients

**Figure 1:**
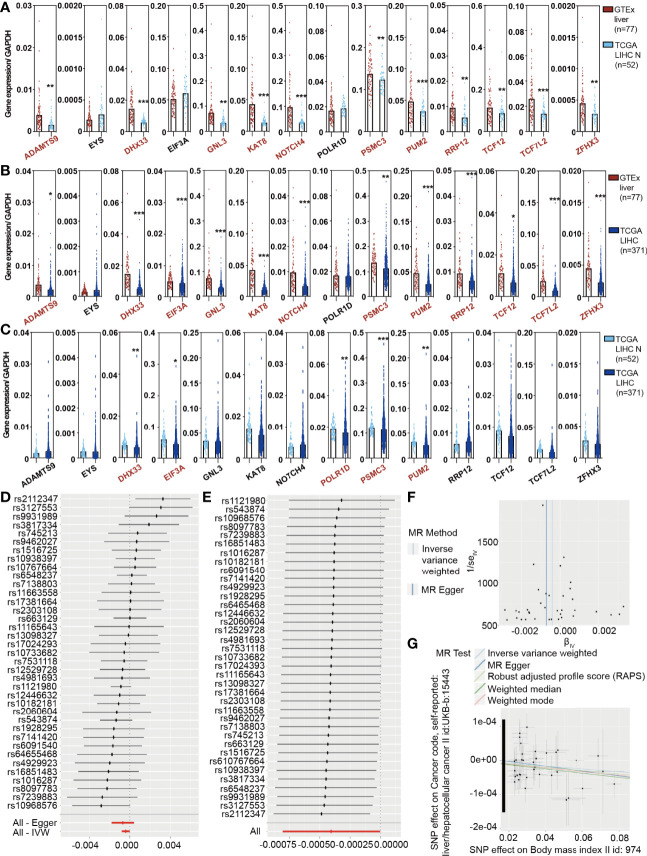
The Cancer Genome Atlas (TCGA) analysis and the MR results reveal a causal association between obesity and hepatocellular carcinoma (HCC) in females. **(A)** Expression of 14 obesity-related genes/GAPDH in GTEx liver and TCGA liver hepatocellular carcinoma (LIHC) normal samples. **(B)** Expression of 14 obesity-related genes/GAPDH in GTEx liver and TCGA LIHC tumor samples. **(C)** Expression of 14 obesity-related genes/GAPDH in TCGA LIHC normal and tumor samples. **(D)** A stratified analysis demonstrated that obesity was not related to the incidence of HCC in the total population. **(E)** A stratified analysis further revealed that obesity was negatively correlated with the incidence of HCC in females. **(F, G)** The Mendelian randomization Egger test showed a significantly negative correlation between obesity and HCC incidence.

To analyze the trend of obesity-associated genes in patients, we calculated the expression of 14 genes/GAPDH in GTEx normal, TCGA LIHC normal, and TCGA LIHC patients to determine whether obesity changed with HCC occurrence. The expression of 14 genes/GAPDH decreased as the disease progressed (**Figures 1A–C**), including *ADAMTS9*, *DHX33*, *EIF3A*, *KAT8*, *NOTCH4*, *PSMC3*, and *PUM2*. The gender of GTEx normal was stratified according to our manual (**Supplementary Figure S1**) (18). Moreover, the changes in 14 genes/GAPDH expressions in female patients are presented in **Supplementary Figure S2**. To study the effect of obesity genotype on the occurrence of HCC, we used the Genome-Wide Association Study (GWAS) database (http://atlas.ctglab.nl/) to search for obesity-related (BMI >25.0 kg/m^2^, body mass index II id: 974) and HCC-related (liver/hepatocellular cancer II id: UKB-b 15443) SNPs and to analyze the correlation between obesity and HCC by MR. Obesity is suggested not to be a risk factor affecting the occurrence of HCC in the general population. A further stratified analysis based on sex found that obesity is related to the incidence of HCC in female patients (**Figures 1D, E**). The MR Egger test showed a significantly negative correlation between obesity in females and HCC incidence (*P* < 0.001, **Figures 1F, G**). This finding indicated that, from a genetic perspective, obesity may be a protective factor for HCC in female patients.

### *MMAA* Is a Differential Obesity Gene and Predicts Better Prognosis in HCC

**Figure 2:**
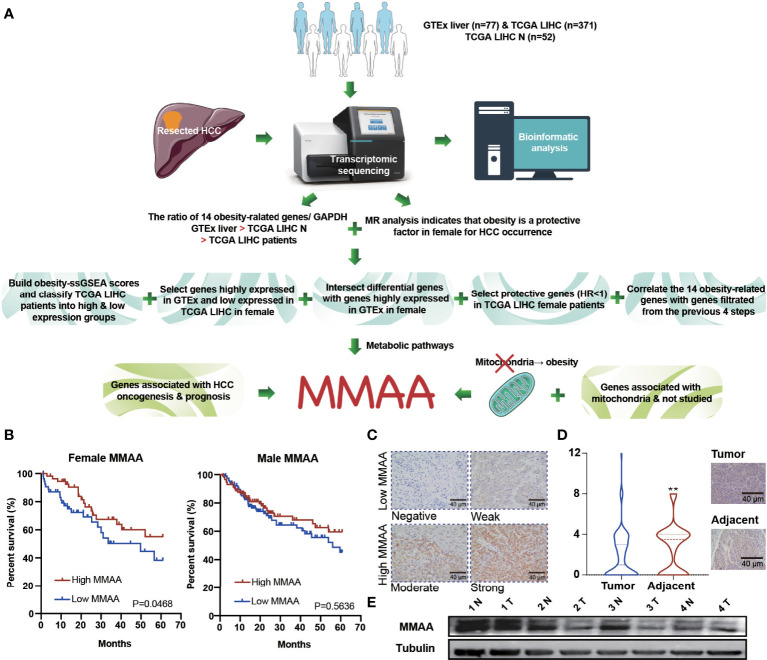
The obesity differential gene MMAA is a positive indicator in the prognosis of HCC. **(A)** A flowchart elucidating the screening criteria for obesity differential genes. **(B)** MMAA was associated with better outcomes in female HCC patients but not in male HCC patients. **(C)** Immunohistochemical staining of MMAA in HCC patients in terms of negative, low, moderate and high expression. **(D, E)** MMAA was highly expressed in adjacent tissues compared with tumoral tissues in HCC (scale bar, 200 µm and 40 µm). (*P < 0.05, **P < 0.01, ***P < 0.001; three biological repeats per group.)

To verify whether obesity has a positive impact on the prognosis of patients with HCC, we illustrated a flow chart to specify our screening criteria. First, we included normal liver samples from the GTEx database (*n* = 77) and 371 LIHC patients from the TCGA database. Second, we found that obese female patients benefited from HCC prevalence *via* MR analysis. Third, we designed the following criteria to select metabolism-related genes: (1) we built obesity-ssGSEA scores and classified TCGA LIHC patients into high -and low-expression groups, (2) we selected genes highly expressed in GTEx and weakly expressed in TCGA LIHC in female patients, (3) we intersected differential genes with genes highly expressed in GTEx in female patients, (4) we selected protective genes (HR <1) in TCGA LIHC female patients, and (5) we correlated at least 12 of the 14 obesity-related genes with genes filtered from the previous 4 steps. Finally, considering that mitochondrial dysfunction is closely related to obesity, we combined the requirements of genes associated with HCC oncogenesis and prognosis as well as mitochondrial function (**Figure 2A**). *MFN2*, *ODGH*, and *MMAA* were eventually selected for survival analysis, but only *MMAA* predicted better 5-year survival outcomes in female HCC patients (**Figure 2B** and **Supplementary Figure 3**; generalized Wilcoxon analysis, *P* = 0.0468). To ensure that the clinical characteristics would not interfere with the conclusion, we analyzed the baseline clinical data of the HCC patients in TCGA (**Supplementary Table S1**). To verify the role that MMAA might play in HCC, we collected 55 tissue sections from HCC patients in our center to measure the expression of MMAA. The clinicopathological features of HCC patients are presented in **Supplementary Table S2**. MMAA was mainly located in the cytoplasm and was more highly enriched in adjuvant tissues than in tumoral tissues (**Figures 2C, D**). Consistently, the protein level of MMAA detected in four patients by Western blotting also supported the immunohistochemical (IHC) staining result (**Figure 2E**). This indicated that MMAA might play an antioncogenic role in HCC, as a high expression of MMAA predicted a superior outcome but still needs further validation.

### *MMAA* Inhibits Cell Proliferation and Migration in HCC

**Figure 3:**
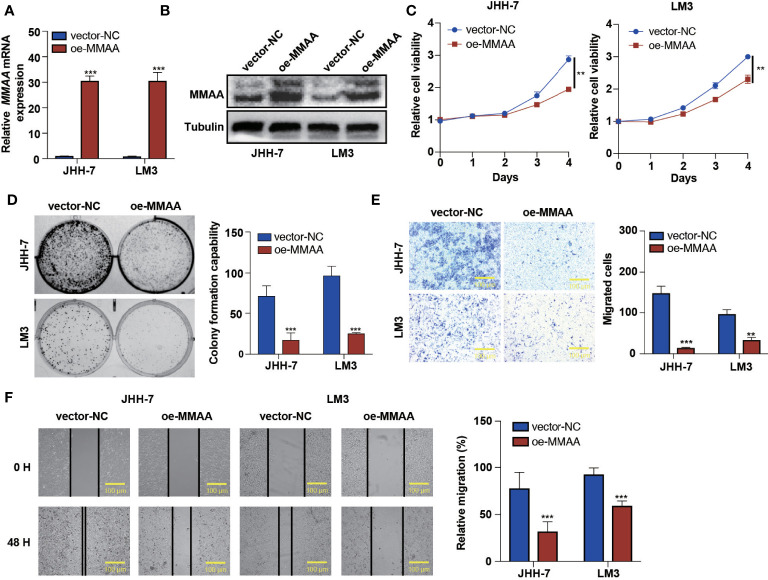
The loss of metabolism of cobalamin-associated A (MMAA) is required for cell proliferation and migration in hepatocellular carcinoma (HCC) *in vitro*. **(A)** Analysis of the relative gene expression data for MMAA using qRT-PCR. **(B)** Analysis of MMAA protein expression using a Western blotting assay. **(C)** An MTS assay was used to detect the proliferation of HCC cells transfected with OE-*MMAA*. **(D)** A colony formation assay was conducted to confirm the effect of MMAA overexpression in two different cell lines, and quantification of the data is shown. **(E)** The cell migration analysis following MMAA overexpression and quantification of the data is shown (scale bar, 100 µm). **(F)** OE-*MMAA* JHH-7 and LM3 cells both exhibited significantly decreased cell motility in the wound healing assay, and quantification of the data is shown (scale bar, 100 µm). **P < 0.01, ***P < 0.001; three biological repeats per group.

Based on the high expression of MMAA in nontumoral tissues and its representativeness in differential obesity genes, we hypothesized that MMAA might influence cancer cell proliferation, colony formation ability, migration, and apoptosis. We first explored the expression levels of MMAA in different HCC cell lines (**Supplementary Figure S4**). Next, we established stably transfected JHH-7 and LM3 cells with a pLVX-FLAG-puro-*MMAA* plasmid, and the overexpression efficiency was confirmed (**Figures 3A, B**). The cell proliferation rate was significantly inhibited in OE-*MMAA* cells (**Figure 3C**). Colony formation was decreased in the OE-*MMAA* group (**Figure 3D**), suggesting that MMAA plays a significant role in suppressing HCC cell proliferation and tumorigenesis. To identify whether MMAA has the potential to affect liver cancer cell mobility, we next performed Transwell assays and wound healing assays in vector-*NC* and OE-*MMAA* cells. OE-*MMAA* JHH-7 and LM3 cells both exhibited reduced numbers of migrating cells in the Transwell assays (147 *vs*. 14 and 97 *vs*. 33, both *P <*0.001, **Figure 3E**). Coinciding with the results of the Transwell assays, the extent of wound closure was greatly inhibited by *MMAA* overexpression (78 *vs*. 32%, 93 *vs*. 60%, both *P <*0.001, **Figure 3F**). Moreover, we validated the tumor biological function of MMAA in SK-Hep-1 cells by transfecting siMMAA, which also strongly suggested that MMAA acted as a tumor suppressor gene that could inhibit the tumor biological behavior of HCC cells (**Supplementary Figures S5A–C, S6A, B, S7A, B**).

### Overexpression of *MMAA* Negatively Regulates Mitochondrial Respiration in HCC

**Figure 4:**
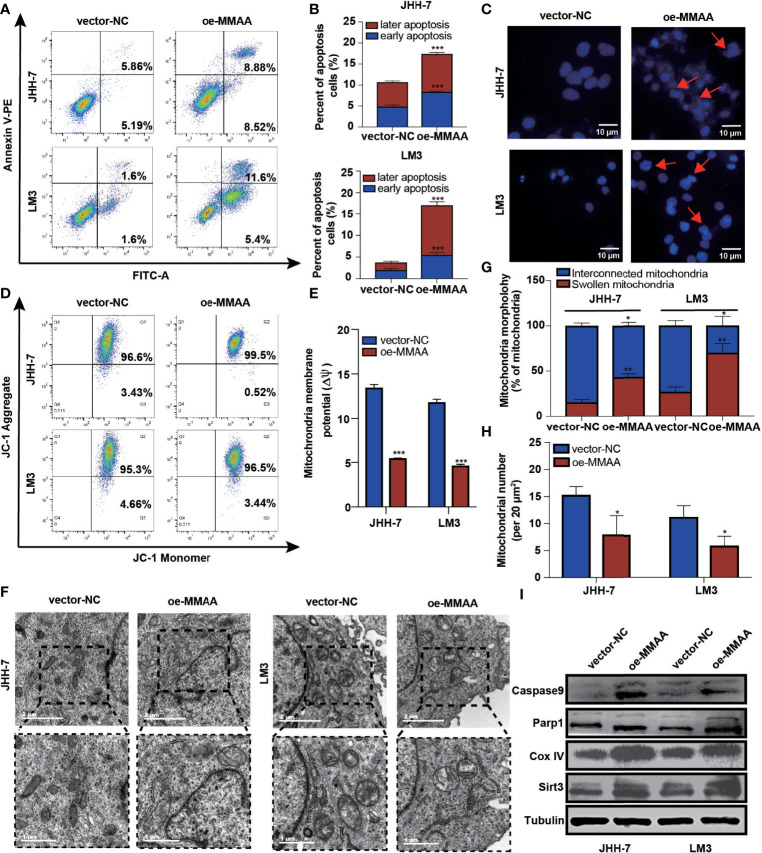
The loss of metabolism of cobalamin-associated A (MMAA) is required for cell proliferation and migration in hepatocellular carcinoma (HCC) *in vitro*. **(A)** Apoptosis rates of the MMAA-overexpressing cell lines compared with vector-*NC*; quantification of the data is presented in **(B)**. **(C)** Increased apoptosis in OE-*MMAA* HCC cell lines was accompanied by shrinkage of the nucleus morphology (scale bar, 10 µm). **(D)** Mitochondrial potential was measured by detecting the JC-1 decrease in JHH-7 and LM3 cells overexpressing MMAA. The decrease in mitochondrial potential was quantified in **(E)** as mean ± SD. **(F)** TEM analysis of the mitochondrial morphology after a stable infection with MMAA in JHH-7 and LM3 cells (scale bars, 2 and 1 µm). **(G)** Quantification of the percentages of swollen mitochondria and interconnected mitochondria through TEM data. **(H)** Quantification of the number of mitochondria per 20 mm^2^ *via* TEM data. **(I)** Immunoblot analysis of mitochondria-related protein expression in JHH-7 and LM3 cells infected with MMAA. *P < 0.05, **P < 0.01, ***P < 0.001; three biological repeats per group.

It is generally perceived that HCC has a high mitochondrial load and that dysfunctional mitochondria account for more than 75% of the cell volume (19). Considering that *MMAA* is involved in the translocation of cobalamin into the mitochondrion, we started to explore whether it participated in the mitochondrial function of energy metabolism reprogramming. We first examined apoptotic changes, which are often mediated by ROS, causing the opening of the mitochondrial membrane permeability transition channel, and the mitochondrial membrane system undergoes rupture and can cause cell death. **Figures 4A, B** show that *MMAA* acted as an apoptosis-promoting molecule, especially in early apoptosis. The overexpression of *MMAA* increased the nuclear density, and the depth of staining and the expression of caspase-9 and Parp1 were also upregulated (**Figure 4I**), indicating the promotion of apoptosis (**Figure 4C**). Correspondingly, the process of cell apoptosis is often accompanied by the destruction of mitochondrial transmembrane potential. By measuring the mitochondrial transmembrane potential *via* JC-1 dye, we found that *MMAA* overexpression decreased the membrane potential of JHH-7 and LM3 cells (**Figures 4D, E**). We continued to explore the changes in the ultrastructure of mitochondria in vector-*NC* and OE-*MMAA* cells through transmission electron microscopy (TEM). Consistent with the abovementioned results, the OE-*MMAA* cells demonstrated more swollen and rounded mitochondria and fewer interconnected mitochondria than the vector-*NC* cells (**Figure 4F**). The number of mitochondria decreased with the overexpression of *MMAA*, indicating that *MMAA* is a vital negative regulator of mitochondrial function (**Figures 4G, H**). In addition, we found that the overexpression of *MMAA* compensatorily increased the expression of two mitochondrion-associated proteins, Sirt3 and COX IV (**Figure 4I**).

### Overexpression of *MMAA* Changes the Metabolic Phenotype in HCC Cells *In Vitro*

**Figure 5:**
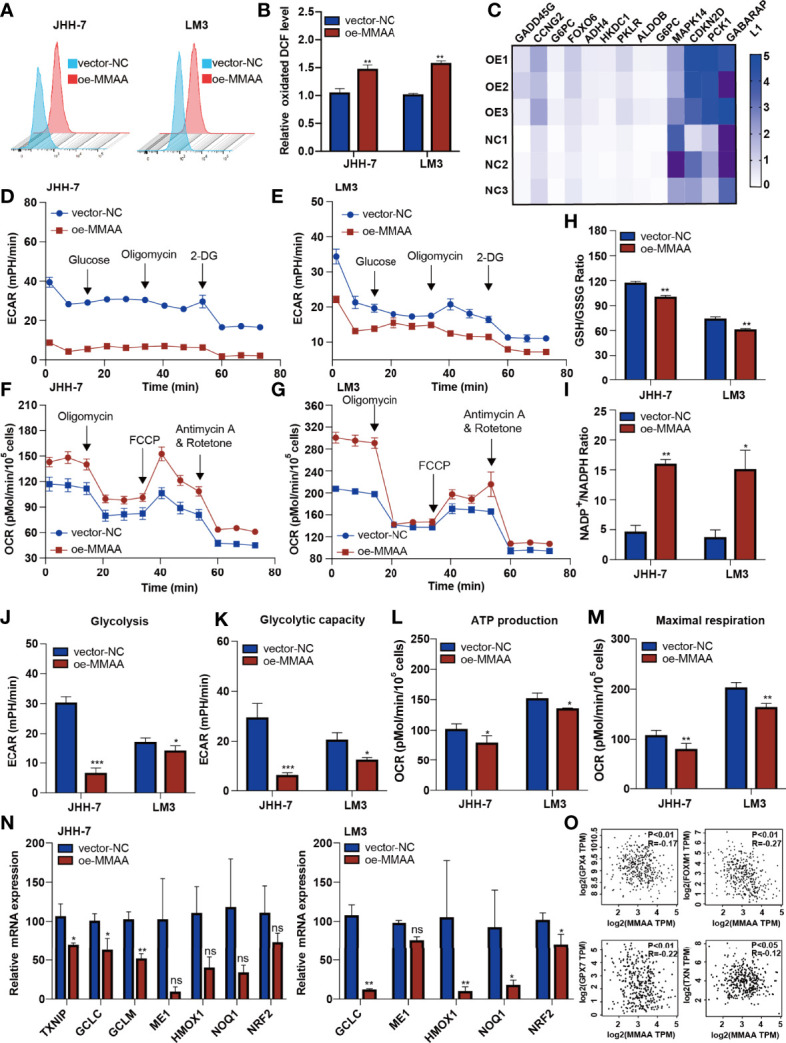
Metabolism of cobalamin-associated A (MMAA) regulates redox balance to alter the mitochondrial function in hepatocellular carcinoma (HCC). **(A)** Reactive oxygen species (ROS) production was measured by flow cytometry in JHH-7 and LM3 cells with an overexpression of MMAA; quantification of ROS production is shown in **(B)**. **(C)** PCR array analysis of the effect of MMAA overexpression on changes in glycolysis and redox state related to mitochondrial function. **(D, E)** Overexpression of MMAA decreased the extracellular acidification rate in JHH-7 and LM3 cells; quantification of glycolysis and glycolytic capacity is shown in **(J, K)**. **(F, G)** Overexpression of MMAA increased the O_2_ consumption rate in JHH-7 and LM3 cells; quantification of ATP production and maximal respiration is shown in (**L**) and **(M)**. **(H)** Determination of the GSH/GSSG ratio in JHH-7 and LM3 cells overexpressing MMAA. **(I)** Determination of the NADP^+^/NADPH ratio in JHH-7 and LM3 cells overexpressing MMAA. **(N)** Quantitative PCR was performed to confirm that the mRNA expression of antioxidant response element-related genes was downregulated by an overexpression of MMAA. **(O)** Pearson’s correlation analysis of different genes expressed in HCC patients’ tissues in the GEPIA2 dataset; *MMAA* expression was negatively correlated with *GPX4*, *FOXM1*, *GPX7*, and *TPM* (*n* = 369). *P < 0.05, **P < 0.01, ***P < 0.001; three biological repeats per group. ns P > 0.05.

The abnormalities of mitochondrial metabolism led to sorafenib resistance in targeted therapy for HCC (20). Additionally, as the permeability pores of the inner mitochondrial membrane of the abnormal mitochondria and the respiratory electron transport chain released ROS, we observed that the *MMAA* overexpression increased the ROS release (**Figures 5A, B**). We next explored the possible mechanism underlying the changes in metabolic phenotype through RNA sequencing. **Figure 5C** shows that genes related to “glycolysis/gluconeogenesis” and “FoxO signaling pathway” were expressed distinctively in the OE-*MMAA* and vector-*NC* groups.

As energetic metabolism is usually altered from mitochondrial respiration to aerobic glycolysis, we measured the extracellular acidification rate (ECAR) and O_2_ consumption rate (OCR) to determine the metabolic phenotype in HCC cells. We found that the overexpression of *MMAA* reduced the glycolysis rate of HCC cells. The basal glycolysis and compensatory respiration were reduced in HCC cells with lentiviral transduction of OE-*MMAA* compared with vector-*NC* (**Figures 5D, E, J, K**). Moreover, the mitochondrial respiration of HCC cells also showed a decreasing trend with the overexpression of *MMAA*. As shown in **Figures 5F, G**, the basal OCR, ATP-linked respiration, maximal OCR level, and spare respiratory capacity were all weakened in OE-*MMAA* cells compared with vector-*NC* cells (**Figures 5L, M**). To some extent, the reduction in ATP consumption means that the energy needed for tumor growth is reduced, and the malignancy of the tumor is decelerated (**Figure 5L**). Considering that the cellular redox balance interferes with mitochondrial respiratory function, we continued to detect the redox level in HCC cells to determine whether *MMAA* could act as an antioxidative molecule. As expected, the GSH/GSSG ratio was decreased in the OE-*MMAA* group in the JHH-7 and LM3 cell lines (**Figure 5H**). Consistently, the NADP^+^/NADPH ratio was also increased in the OE-*MMAA* group compared with the vector-*NC* group (**Figure 5I**). These results confirmed our hypothesis that *MMAA* overexpression could influence the redox balance and that antioxidative genes, such as *TXNIP*, *GCLC*, *GCLM*, *HMOX1*, *NOQ1*, and *NRF2*, were downregulated in the OE-*MMAA* group (**Figure 5N**). In validating whether the expression of MMAA could affect the expression of antioxidative genes in large population samples, we found that the expression of MMAA was negatively correlated with the antioxidative genes (*GPX4*, *FOXM1*, *GPX7*, and *TNX*) in TCGA (**Figure 5O**). This indicated that antioxidative genes disturbed the unfavorable effect of *MMAA* on mitochondrial function in HCC.

### *MMAA* Overexpression Inhibits Hepatic Xenograft Tumors From Proliferating

**Figure 6:**
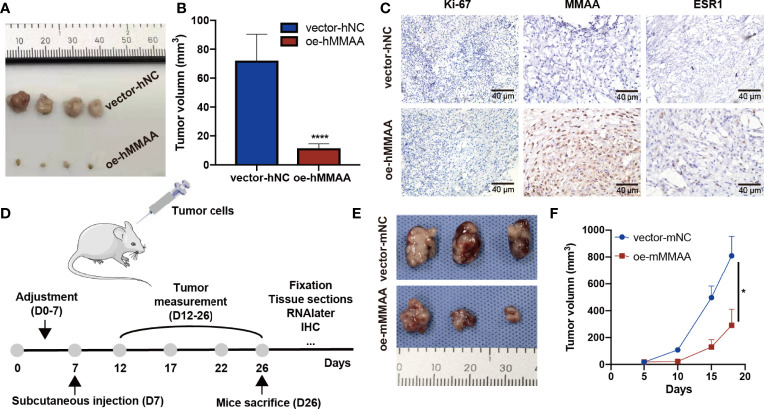
Metabolism of cobalamin-associated A (MMAA) inhibits hepatocellular carcinoma (HCC) progression in xenograft models and is positively correlated with ESR1 *in vivo*. **(A)** Stably infected OE-*MMAA* or vector-*NC* HCC cells were injected into nude mice and measured every 3 days. **(B)** The tumor sizes were calculated and compared during the day of resection. **(C)** The tissue sections were stained for Ki-67, MMAA, and ESR1, and a positive correlation was found between ESR1 and MMAA (scale bar, 40 µm). **(D)** To verify whether MMAA can indeed inhibit the growth of HCC, we constructed a murine OE-*MMAA* HCC cell line and established xenograft models following the procedure. **(E)** The tumor sizes were measured by electronic Vernier calipers in the OE-m*MMAA* and vector-m*NC* groups. **(F)** Tumor growth curves were created based on the tumor volume. *P < 0.05, ****P < 0.001; ≥3 biological repeats per group.

To further clarify the inhibitory function of MMAA in HCC, xenograft tumors were established in both a human HCC cell line and a murine cell line. As presented in **Figures 6A, B**, the tumor sizes were significantly smaller in the OE-*hMMAA* group than in the control group. More importantly, Ki-67 expression was reduced in the OE-*hMMAA* group accordingly (**Figure 6C**) in HepG2 cells. Furthermore, we found that the expression of ESR1 was also positively correlated with the expression of MMAA (**Figure 6C** and **Supplementary Figure S8**), suggesting that *MMAA* might be regulated by its upstream regulator *ESR1*. The ability of anticancer progression was also validated in murine xenografts (**Figures 6E, F**) following the implantation procedure in **Figure 6D**. This indicated that MMAA could inhibit the proliferation of HCC at the genetic level regardless of the species.

### *MMAA* Is a Downstream Target of *ESR1*

**Figure 7:**
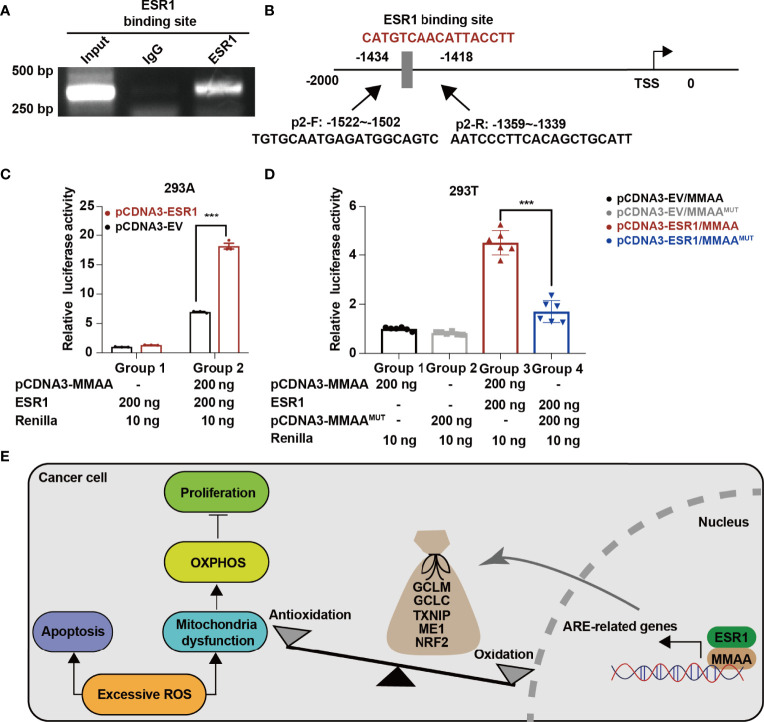
ESR1 regulates the metabolism of cobalamin-associated A (MMAA)/antioxidant response element activation in HCC. **(A)** ESR1 occupied the binding site on the MMAA promoter region as revealed by ChIP assays. **(B)** Position of the potential sequence in the MMAA promoter. **(C, D)** ESR1 increased the luciferase activity of the MMAA promoter in 293A and 293T cells. **(E)** A working model illustrating the antioncogenic function of MMAA in HCC. ***P < 0.001; three biological repeats per group.

As we found that obese women have a favorable prognosis in the development of HCC, we wondered whether the female estrogen receptor and the transcription factor *ESR1* are involved in the activation of *MMAA*. As we found that MMAA was upregulated with the increase in ESR1 expression (**Supplementary Figure S9**), we first conducted a chromatin immunoprecipitation (ChIP) assay to determine whether *ESR1* could bind with the consensus *ESR1* binding element. From **Figure 7A**, we can see that *ESR1* could combine with the consensus *ESR1* binding element in the promoter region starting from -2,000 to 0 of *MMAA* (**Figure 7B**). The luciferase assays illustrated that manipulation of ESR1 expression promoted MMAA promoter activity in 293A cells (**Figure 7C**). To further validate that MMAA is a downstream target of ESR1, we mutated the promoter sequence CATGTCAACATTACCTT into TTCAGTCGTGCGTAATG, and the expression level of *MMAA* failed to be upregulated by *ESR1* (**Figure 7D**).

In conclusion, our findings demonstrated that obese female patients benefit from the incidence of HCC, and the obesity-differentiated gene *MMAA* is a novel predictive marker for better outcomes in HCC. *In vitro* cell lines and *in vivo* experiments showed that the overexpression of *MMAA* negatively regulates cell proliferation, migration, and anaerobic glycolysis. Moreover, *MMAA* is correlated with antioxidative genes in perturbing redox balance and reprogramming the metabolic pattern of HCC by altering the mitochondrial function. Finally, the current study uncovers the ESR1/MMAA axis as a potential therapeutic target for HCC. Further studies are needed to elucidate this axis to improve the overall survival of HCC patients (**Figure 7E**).

## Discussion

Primary HCC is more than two times more prevalent in men than in women, which is also observed in male mice developing liver tumorigenesis *via* diethylnitrosamine compared with the incidence in female patients (21, 22). Clinical findings have demonstrated that the incidence of HCC and the related mortality rates are higher in men than in women (23). In the past few decades, great efforts have been made to identify potential molecular targets that could elucidate the sex dimorphism in HCC but have failed. Herein we found that obesity might protect obese females from HCC and identified *MMAA* as an obesity-differential gene as well as a prognostic index for HCC. *MMAA* overexpression in HCC cells inhibits cancer cell proliferation, migration, and clone formation, increases the apoptosis rate, and maintains the redox balance as a result of mitochondrial dysfunction. Moreover, we demonstrate that *MMAA* is a downstream target of *ESR1*, which further explains why *MMAA* benefits female HCC patient outcomes in the long term.

It is commonly believed that obesity leads to the onset of cancer; however, recent studies have discovered that obesity might not influence all cancer types in the same way as carcinogenesis. The increase in BMI increases the proportion of lipocytes, which produce a large amount of estrogen. It was reported in some studies that whether mice were fed a high-fat food or a normal diet, the probability of the mice developing HCC did not change in the two groups; however, the probability increased when cancer-promoting substances were added, which indicates that obesity is not the direct cause of cancer. Men are more likely to suffer from HCC than women, and ovariectomy increases the incidence of HCC in women, while castration decreases the incidence in men (24, 25). Therefore, ovarian hormones can prevent HCC, and androgens can promote tumor growth. Moreover, studies have shown that estrogen plays a role in the promotion phase of carcinogenesis rather than in the initial phase. Additionally, ESR1 has been proven to suppress proliferation, migration, and invasion in HCC cells, and its underexpression in male patients predicts a worse prognosis in HCC (26). Therefore, regardless of many studies reporting that the incidence of HCC might be related to obesity in a large population, some confounding factors might be neglected, such as serum lipids, sex hormones, blood glucose, and hereditary factors. Genes are randomly assigned to offspring during meiosis, which is equivalent to randomly assigning gene effects to “subjects”, thereby “controlling” the influence of other factors on gene effects. To reveal the real relationship between exposure and outcomes, researchers use genetic variation as an instrumental variable to study the relationship between instrumental variables and outcomes and, from the relationship between the two, to deduce the effect of exposure (gene effect) on the outcome (27). Therefore, to determine whether obesity is the onco-carcinogenesis factor of HCC and whether it affects both sexes equally, MR was applied to excavate the GWAS database (http://atlas.ctglab.nl/) and correlate the SNPs of obesity (BMI > 25.0 kg/m^2^), and the representative SNPs of HCC are a good way to explore the possible underlying mechanisms. Surprisingly, we found that obesity in female patients may be a protective factor for HCC. ESR1 is a ligand-activated transcription factor comprising several domains important for hormone binding, DNA binding, and transcription activation and encodes ER, and it is a substance that promotes the development of secondary sexual characteristics and the maturation of sexual organs in female animals. Adipose tissue produces estrogen, which might indicate an underlying relationship between ESR1 and obesity-related genes (10). Clarifying the obesity-differential genes in HCC patients and specifying their relationship with ESR1 are the key factors to illuminating how obese female patients benefit from HCC oncogenesis. Our results also showed that *ESR1* directly regulates the obesity-differential gene *MMAA* to improve the prognosis of HCC patients.

The tumor environment is greatly affected by BMI in tumoral and peritumoral adipose tissues, and it thereby influences the efficacy of immunotherapy clinically and supplies sufficient energy for cancer cells to flexibly upregulate the free fatty acid pathway, take up more lipids, and indirectly inhibit the tumor infiltration and antitumor function of CD8+ T cells (28, 29). Obesity-related genes might be involved in metabolic reprogramming, which is essential for malignant stemness and cancer cell proliferation. Metabolic abnormalities are one of the crucial characteristics of cancer cells because they undergo a series of metabolic reprogramming to meet their own rapid proliferation needs. The Warburg effect proposed in the 1920s noted that, even under aerobic conditions, tumor cells will maintain anaerobic glycolysis to produce energy and raw materials for cell proliferation and sustain their malignant properties (30). In addition to ATP, Sanchez-Perez *et al.* noted that oxidative stress interferes with DEN metabolism by increasing ROS and oxidized thiol proteins to accelerate the HCC carcinogenesis process, indicating that it might be possible to focus on the development of targeted therapies aimed at disturbing the redox balance (31). As all paths lead to NADPH in cancer, NADP is frequently converted to NADPH by glucose 6-phosphate dehydrogenase and malic enzymes, accompanied by the overexpression of mutant p53 in tumor cells (32). Notably, p53 could partially activate detoxification cytochrome P450 to control ROS and limit HCC progression (33). The main mechanism used by cancer cells to reduce ROS levels relates to the production of glutathione and its application in the reaction H_2_O_2_ + 2GSH → GSSG + 2H_2_O, where GSH is reduced glutathione. Additionally, blocking glutamine metabolism helps inhibit the growth and proliferation of HCC cells by sealing the production and utilization of glutamine metabolites. Therefore, p53 expression interacts with glutamine metabolism to preserve the distinct redox state of HCC (34). NADH+ is an indispensable molecule for mitochondrial function, serving roles in oxidative phosphorylation as well as redox balance (35). Therefore, we hypothesized that HCC cells were trained to develop to protect themselves from respiratory injury to maintain the redox balance.

Once the energy factory—mitochondria—is destroyed, cancer cells grab mitochondria from surrounding healthy cells to restore their functions. We observed that the apoptosis rate was elevated in the OE-*MMAA* group in HCC; the apoptosis rate is often mediated by ROS, causing structural changes, ruptures, and fragmentation of the mitochondrial membrane and resulting in alterations in permeability as well as cell death. The *MMAA* gene is essential for the coding of Cbl reductase and the mitochondrial transporter of vitamin B12 (36). The defect of *MMAA* could lead to the reduction in AdoCbl synthesis located in mitochondria, which also leads to methylmalonic acidemia in clinical practice (37). Watanabe *et al.* discovered an NADPH-dependent aquacobalamin reductase in rat liver from one CblA cell line, further confirming that MMAA protein is mitochondria-targeted (38). Thus, we hypothesized that MMAA could participate in oxidative injury and the biological behavior of mitochondria. Our findings show that the overexpression of *MMAA* could increase the ROS level and restrict mitochondrial function in HCC cells. It is well recognized that, when a cancer suppressor gene is overexpressed, mitochondrial function is recovered, and the glycolysis level is downregulated. However, *MMAA* might be an exception because we identified that *MMAA* balanced the redox status to resist potential oxidative stress. Therefore, we assumed that MMAA was ineluctable for mitochondrial respiration and metabolic reprogramming in HCC.

Due to the heterogeneity of tumor metabolism, different hypoxic conditions, and pH, different types of cancer have distinct sensitivities to oxidative damage. Molecules that disturb the active oxygen scavenging system can also perturb glutamine glycolysis, which inhibits the growth and proliferation of HCC cells (39). NRF2 has been reported to act as an important transcription factor to maintain redox homeostasis, influencing the mitochondrial membrane potential. Additionally, the RNA sequence in our study indicated that overexpression of MMAA altered the genes associated with “glycolysis/gluconeogenesis” and the “FoxO signaling pathway”, which closely influence metabolic reprogramming. We reckoned that the overexpressed MMAA might affect the expression of the antioxidant response element (ARE). The activation of the NRF2-KEAP1 pathway is one of the most important mechanisms for antitumorogenesis, and it can cause the conformational change of KEAP1-CUL3-E3 ubiquitin ligase and subsequently translocate *NRF2* to the nucleus, where it binds to the ARE (40). This would induce the expression of a series of cytoprotective genes, such as *NQO1*, *GST*, *HMOX1*, and *GCLC*. We further confirmed in HCC cell lines that the expression of *TXNIP*, *GCLC*, *GCLM*, and *ME1* was downregulated in the OE-*MMAA* group compared with the control. Moreover, we explored the TCGA database and determined that *GPX4*, *FOXM1*, *GPX7*, and *TXN* were all negatively correlated with the expression of MMAA (*P* < 0.05). Thus, we concluded that *MMAA* might participate in interacting with ARE genes to impair ROS detoxification and disturb mitochondrial function.

Taken together, our study illustrated the possible mechanism by which obese female patients have a better prognosis in HCC. As ESR1 promotes the development of secondary sexual characteristics in female animals, we demonstrate a novel function for it to directly regulate the obesity-differential gene *MMAA* and affect the cancer biological behavior of HCC. Moreover, *MMAA* mediates mitochondrial function and alters metabolic reprogramming by influencing oxidative stress and ARE genes. Thus, the obesity-differential gene *MMAA* is oxidative and plays a specific role as a cancer suppressor, and it provides a certain therapeutic value for HCC patients, especially for female patients.

## Materials and Methods

### Comparison of the Expression Levels of 14 Obesity-Related Genes Among GTEx Liver, TCGA Normal Liver, and Tumor Tissues

The 14 obesity-related genes included *ADAMTS9*, *NOTCH4*, *EYS*, *DHX33*, *EIF6*, *GNL3*, *KAT8*, *POLR1D*, *PSMC3*, *PUM2*, *RRP12*, *TCF12*, *TCF7L2*, and *ZFHX3*. We compared the expression of the 14 genes/GAPDH among GTEx liver, TCGA normal liver, and LIHC tissues. Student’s *t* test was used to compare the ratio of 14 genes/GAPDH between groups. We performed a cluster analysis *via* Python (Version 3.7) to distinguish male and female patients in GTEx based on the expression of two genes (XIST and RPS4Y1) (18). GTEX is a commonly used normal tissue database, but for the first time, we developed a manual to apply unique male and female genes as gender identification markers, and the related information is attached in the **Supplementary Materials**. A single-sample gene-set enrichment analysis (ssGSEA) was conducted based on the expression level of 14 obesity-associated signatures using the R packages “GSEAbase” and “GSVA”. The differential genes generated from high- and low-obesity-score groups based on ssGSEA were extracted (logFC <-0.3) and then intersected with genes highly expressed in female GTEx compared with those in male GTEx (logFC >0.3). After the first intersection, genes highly expressed in female GTEx compared with TCGA LIHC tumors were screened out (logFC >0) and were calculated *via* univariate Cox analyses to select the protective genes in HCC (hazard ratio <1). After the third selection, the remaining 291 genes were correlated with the 14 obesity genes (*R >*0.5; correlated with 12 genes or more as the boundary and *P <*0.05), and those relevant to metabolic pathways were selected. A total of 33 genes were later searched to determine whether they were related to HCC oncogenesis. *MFN2*, *OGDH*, and *MMAA* were chosen, and *MMAA* was the only gene associated with a better prognosis in female HCC.

### Human Tissues and Cell Culture

Clinical tissue samples were obtained from patients who were diagnosed at Sir Run-Run Shaw Hospital (SRRSH) between 2007 and 2012. The current study conformed to the principles of the Declaration of Helsinki and was approved by the Institutional Review Board of the SRRSH. All patients gave written informed consent for the use of their tissues for scientific research. The pathological diagnosis was based on the morphological and immunohistochemical criteria provided by the 8th edition of the American Joint Committee on Cancer (41). JHH-7, LM3, HepG2, Huh7, HA22T, SK-Hep-1, and Hepa1-6 cells were purchased from the American Type Culture Collection and verified by DNA fingerprinting. The culture environment was maintained at 37°C with 5% CO_2_.

### Plasmids

The pLVX-FLAG cloning vector (Addgene) was used to generate OE-*MMAA* constructs, and the 41 base pairs (bp) targeted against MMAA were CTGTTCCAGGGGCCCACCGGTCCCATGCTGCTACCACATCC and CTCCAGCGAATTGGCGAATTCTTAGTCTCTGCTTTTAAAAGCTTTTAACA. Overexpression lentivirus particles were produced by cotransfecting lentivirus constructs with pMD2.G and psPAX2 in a 3:2:1 ratio and then added to HEK-293T cells. The pLVX-FLAG vector was designed as a control plasmid.

### Quantitative Real-Time PCR Detection and Quantitative Analysis

Total RNA was isolated using an RNA-Quick Purification Kit (ES Science, China) and was assessed for quality and quantity using absorption measurements. RNA was then reverse-transcribed to cDNA using an Evo M-MLV RT Premix kit (Accurate Biology, China). The cDNA was added to Hifair™ qPCR SYBR Green Master Mix (Yeasen, China) to form a mixture, and then the expression levels of the candidate genes were determined using an ABI 7900HT Real-Time PCR system (Applied Biosystems, USA). A comparative data analysis was performed *via* the 2^-ΔΔCt^ method using the PCR Array Data Analysis web portal (http://gncpro.sabiosciences.com/gncpro/gncpro.php) to determine relative expression differences between the comparison groups. All reactions were run in triplicate, and the primer sequences are listed in **Supplementary Table S3**.

### Animal Model

A xenograft mouse model was used to verify the antioncogenic function of MMAA *in vivo* in both human and murine HCC cell lines. BALB/c-nu female mice, 4 weeks of age, were housed in sterile and filter-capped cages for 1 week before subcutaneous tumor implantation. The mice were randomly divided into two groups (*n* = 3 or 4/group) and injected with 100 μl phosphate-buffered saline (PBS) containing 3 × 10^6^ HepG2 (human)/Hepa1-6 (murine) OE-*MMAA* cells or vector-*NC* type. After 1 week of tumor formation, the sizes of the xenografts were measured twice a week, and the volume was determined using the formula: volume = length × width^2^/2. All tumor specimens were harvested after 3 to 4 weeks of tumor implantation and fixed with 4% paraformaldehyde before being sectioned into tissue slices for IHC staining. All animal experiments were conducted with consent from the Committee of the Use of Live Animals in Teaching and Research at SRRSH.

### Western Blotting

Western blotting was performed as previously described (42). The antibodies used in the current study were anti-MMAA antibodies (Novus Biologicals, USA; Abcepta, China), anti-ESR1α antibody (Abcam, USA), anti-caspase-9 antibody (Abclonal, China), anti-PARP1 antibody (Abclonal), anti-β actin antibody (Proteintech, USA), and anti-α tubulin antibody (Proteintech).

### Cell Proliferation and Colony Formation Assay

Cell proliferation was detected by the MTS assay. A total of 2 × 10^3^ cells were seeded into 96-well plates. MTS Reagent (Abcam) was added to the plates every day, and the cells were cultured for an additional 2 h. The absorbance values were measured at a wavelength of 490 nm by a SpectraMax M2e microplate reader (Molecular Devices, USA). In the colony formation assay, 1 × 10^3^ cells were seeded into 6-well culture plates. The colonies were fixed in 4% paraformaldehyde, stained with crystal violet solution (Beyotime Biotechnology, China), and counted under a microscope after 2 weeks.

### Chromatin Immunoprecipitation Assay

ChIP was performed using a SimpleChIP^®^ Enzymatic Chromatin IP Kit (Cell Signaling Technology, USA) according to the manufacturer’s protocol. In brief, Hep3B cells were cross-linked with formaldehyde and then lysed *via* SDS lysis buffer. Total nuclear DNA was sheared by enzymatic digestion with micrococcal nuclease. Protein–DNA complexes were precipitated by ESR1α antibody or normal rabbit IgG as a negative control. After elution of the complex from the antibody, PCR analysis was performed with primers specific for the *MMAA* promoter. The primers for the P1, P2, and P3 fragments are listed in **Supplementary Table S4**.

### ROS Evaluation

The cells were stained with H2DCF-DA provided in the ROS Assay Kit (Beyotime Biotechnology) according to the manufacturer’s instructions. Cells were washed, resuspended in ice-cold PBS, and collected. The fluorescence intensities of dichlorofluorescein (DCF) formed by the reaction of DCF with ROS were detected by a FACScan flow cytometer (BD Biosciences, USA) at an excitation wavelength of 488 nm and an emission wavelength of 530 nm.

### Evaluation of the Intracellular Reduced Glutathione Oxidized/Glutathione Ratio and NADP^+^/NADPH Ratio

According to the manufacturer’s protocol, a GSH/GSSG Ratio Detection Assay Kit (Elabscience, China) was used to measure the intracellular GSH/GSSG ratio, with an absorbance at 412 nm. The NADP^+^/NADPH Assay Kit (Elabscience) was used to determine the NADP^+^/NADPH ratio. Both analyses were performed to measure the oxidation state of HCC cells.

### IHC

IHC was used to evaluate the expression of MMAA in tumoral and normal hepatic tissues. After being processed for paraffin embedding, 5-μm sections of tissue samples were prepared. First, these sections were deparaffinized and rehydrated. For antigen retrieval, the sections were immersed in 10 mM citrate buffer (pH 6.0) and boiled for 10 min in a microwave oven. Then, endogenous peroxidase activity was blocked with 3% hydrogen peroxide for 10 min. Nonspecific binding sites were blocked with 5% normal goat serum for 30 min. The sections were incubated with an antibody against MMAA (1:1,000, Novus) overnight at 4°C. The sections were then incubated with the secondary antibody, and the expression of MMAA in the tissues was observed *via* microscopy after diaminobenzidine staining and hematoxylin staining.

### Dual-Luciferase Reporter Assay

The *MMAA* promoter region was obtained from the UCSC Genome Database (https://genome.ucsc.edu/), spanning from -2,000 to 0 at the transcription start sites, and was amplified from genomic DNA using the following primers: (forward) ATTTCTCTATCGATAGGTACCTGCTAGCAGTGAGCAAGGCTC and (reverse) ACTTAGATCGCAGATCTCGAGAAATCCCCAATGTGACAGTGTTG). Analysis of putative transcription factor-binding sites on the *MMAA* promoter was performed using JASPAR (https://jaspar.genereg.net/). The wild-type (CATGTCAACATTACCTT) or mutant (TTCAGTCGTGCGTAATG) seed binding site of the MMAA promoter region was subcloned into the pGL3 luciferase reporter vector and verified by sequencing. HEK-293T cells were transfected with the wild-type or mutant reporter construct pRL-TK plasmid, and the luciferase activities were detected using a dual-luciferase system (Promega, USA) based on the manufacturer’s protocol 36 h later.

### Detection of Mitochondrial Membrane Potential

The mitochondrial membrane potential (Δψm) in cardiomyocytes was assessed using 5,5′,6,6′-tetrachloro-1,1′,3,3′-tetraethylbenzimidazolyl-carbocyanine iodide (JC-1) (Beyotime Biotechnology). Briefly, cells were incubated in 6-well plates after the indicated treatments, and equal volumes of JC-1 staining solution (5 μg/ml) were added, incubated at 37°C for 20 min, and washed twice with PBS. Cells were analyzed using a multilabel plate reader (PerkinElmer, USA) with 488-nm excitation, 530-nm emission for green, and 590-nm emission for red. Alteration in the ionic equilibrium results in mitochondrial depolarization. Mitochondrial depolarization was indicated by an increase in the red/green fluorescence intensity ratio.

### Extracellular Acidification Rate and Oxygen Consumption Rate

Cellular mitochondrial function and glycolytic capacity were measured using the Seahorse Bioscience XF96 Extracellular Flux Analyzer according to the manufacturer’s instructions of the Seahorse XF Cell Mito Stress Test Kit or Glycolysis Stress Test Kit (Seahorse Bioscience, USA). JHH-7 and LM3 cells were plated in XF96 Cell Culture Microplates (Seahorse Bioscience) at an initial cellular density of 4 × 10^4^ cells/well the day before determination. Then, we washed the cells with Seahorse buffer containing 175 μl of Seahorse buffer plus 25 μl each of 1 mmol/L FCCP [carbonyl cyanide-4-(trifluoromethoxy)phenylhydrazone] and 1 mmol/L oligomycin, and 1 mmol/L rotenone was automatically injected to measure the OCR. For ECAR measurement, 10 mM glucose, 1 μM oligomycin, and 100 mM 2-deoxy-glucose were automatically added to measure the ECAR value. The OCR and ECAR values were calculated after normalization to the cell numbers and were plotted as mean ± SD.

### Measurement of Cell Apoptosis

FITC Annexin V Apoptosis Detection Kit (Multi Sciences, China) was used to measure the apoptotic rate. Cells were analyzed according to the manufacturer’s instructions and counted using a FACSCalibur flow cytometer.

### Statistics

SPSS software (version 22.0, IBM Corp., USA) was used for the data analysis, and Student’s *t*-tests were applied for most occasions. The experiments were repeated at least three times and are presented as mean ± SD. One-way ANOVA was used to analyze the differences among three or more groups. Statistical differences were regarded as significant at *P <0.05, **P <0.01, and ***P <0.001.

## Data Availability Statement

The original contributions presented in the study are included in the article/**Supplementary Material**. Further inquiries can be directed to the corresponding authors.

## Ethics Statement

This study was approved by the Institutional Review Board of the SRRSH (ethical code: 20200210-126) and written informed consent was obtained from all patients. All of the animal experimental protocols were approved by the Committee of the Use of Live Animals in Teaching and Research at SRRSH (ethical code: SRRSH20210130).

## Author Contributions

Conceptualization: YZ, QM, and CZ. Methodology: YZ, JC, and QM. Investigation: YL, QX, PC, and CZ. Visualization: YL and XF. Supervision: DH, QM, and HL. Writing—original draft: YZ and JXC. Writing—review and editing: YZ, CZ, HL, and DH. All authors contributed to the article and approved the submitted version.

## Funding

This work was supported by the National Key Research and Development Project (2017YFC0110802), Zhejiang Province Key Research and Development Project (2020C01059), National Natural Science Foundation of China (81872297, 81874059, and 82102105), Zhejiang Engineering Research Center of Cognitive Healthcare (2017E10011), National Key Scientific Instrument and Equipment Development Project (81827804), and China Postdoctoral Science Foundation (2021M702825).

## Conflict of Interest

The authors declare that the research was conducted in the absence of any commercial or financial relationships that could be construed as a potential conflict of interest.

## Publisher’s Note

All claims expressed in this article are solely those of the authors and do not necessarily represent those of their affiliated organizations, or those of the publisher, the editors and the reviewers. Any product that may be evaluated in this article, or claim that may be made by its manufacturer, is not guaranteed or endorsed by the publisher.
