# Supplementary material for: ESR1 Regulates the Obesity- and Metabolism-Differential Gene MMAA to Inhibit the Occurrence and Development of Hepatocellular Carcinoma
Source: Front Oncol. 2022 Jun 20;12:899969. doi: 10.3389/fonc.2022.899969 (PMC9252523; doi:10.3389/fonc.2022.899969)
Supplement: Supplementary file 1 [file DataSheet_1.docx]

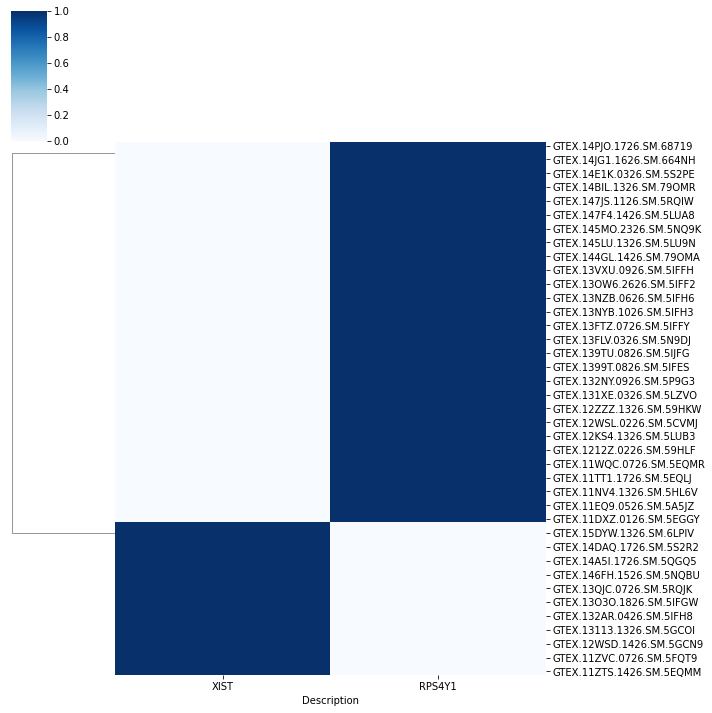


**Figure S1. *XIST* and *RPS4Y1* were used to distinguish between women and men in GTEx liver database.**

**
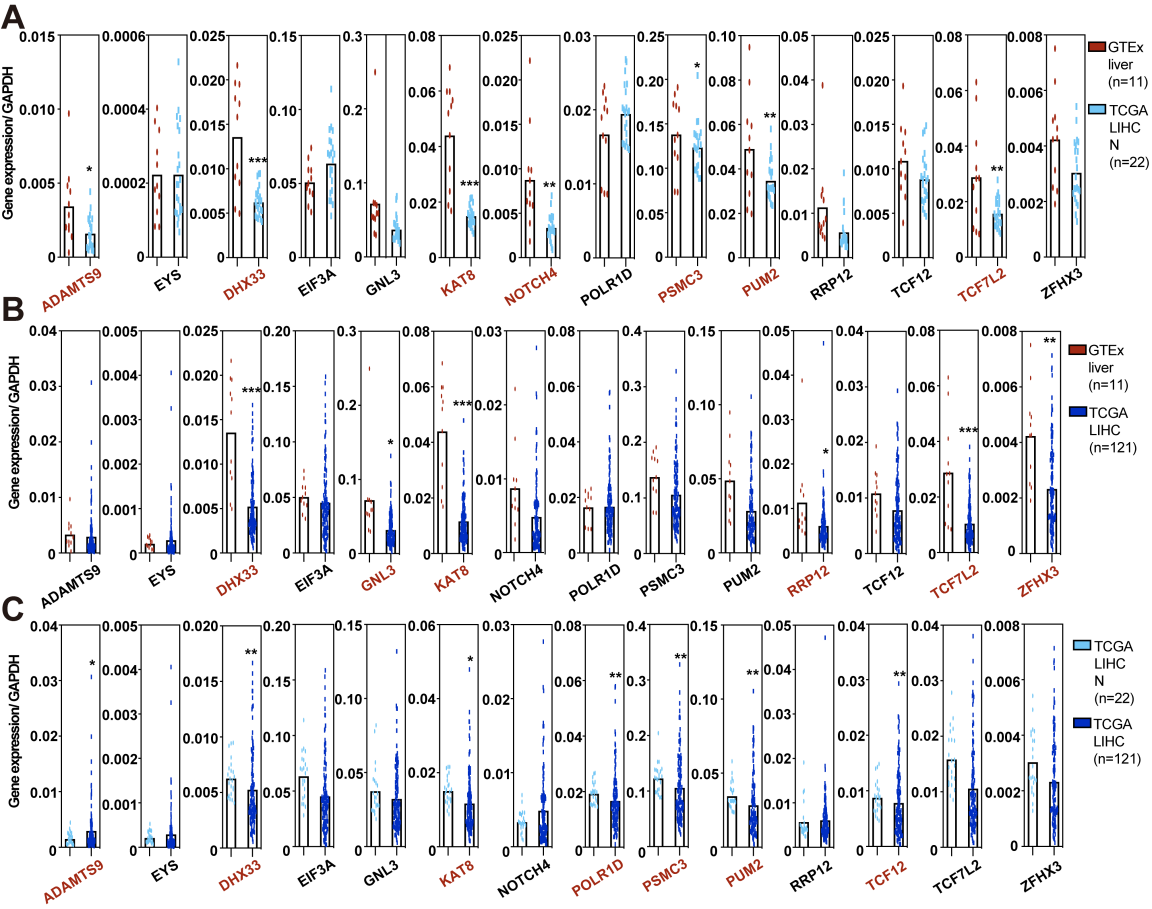
**

**Figure S2. The expression of 14 obesity-related genes/ GAPDH in GTEx liver, TCGA LIHC normal samples and TCGA LIHC tumor samples in female.** (A) The expression of 14 obesity-related genes/ GAPDH in GTEx liver and TCGA LIHC normal samples in female. (B) The expression of 14 obesity-related genes/ GAPDH in GTEx liver and TCGA LIHC tumor samples in female. (C) The expression of 14 obesity-related genes/ GAPDH in TCGA LIHC normal and tumor samples in female. (*P < 0.05, **P < 0.01, ***P < 0.001.)

**Figure S3. Thirty-three genes were screened out to explore the relationship with the occurrence and development of HCC.** ***MFN2*, *OGDH* and *MMAA* were key proteins related to mitochondria, and the 5-year survival curves were generated in MFN2 and OGDH in female and male respectively.**

**Figure S4. The expression of MMAA in different HCC cell lines.**

**Figure S5. Decreased expression of MMAA could accelerate HCC cells to proliferation.** (A) Analysis of relative gene expression data for MMAA using qRT–PCR. (B) ﻿Analysis of MMAA protein expression using a Western blotting assay. (C) ﻿﻿An MTS assay was used to detect the proliferation of HCC cells transfected with siMMAA. (*P < 0.05 , ***P < 0.001; ≥ three biological repeats per group.)

**Figure S6. Decreased expression of MMAA could promote HCC cells to form colonies.** (A) ﻿A colony formation assay was conducted to confirm the effect of MMAA downregulation in SK-Hep-1, and quantification of the data is shown in (B). (**P < 0.01; ≥ three biological repeats per group.)

**Figure S7. Decreased expression of MMAA could accelerate HCC cells to migrate.** (A) ﻿The cell migration analysis following MMAA downregulation and quantification of the data is shown (scale bar, 100 µm). (B) siMMAA SK-Hep-1 cells exhibited significantly increased cell motility in the wound healing assay, and quantification of the data is shown (scale bar, 100 µm). ﻿(**P < 0.01, ﻿***P < 0.001; ≥ three biological repeats per group.)


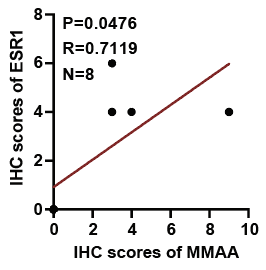


**Figure S8. The expression of ESR1 was positively correlated with the expression of MMAA in IHC scores.**


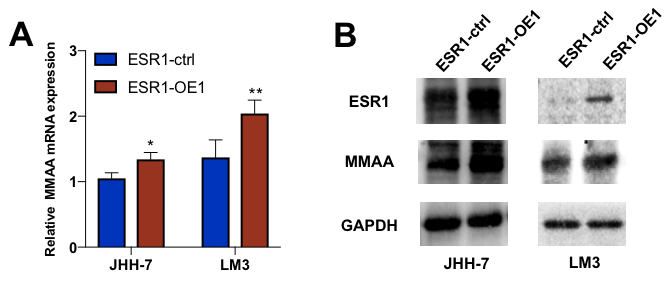


**Figure S9. MMAA was upregulated with the overexpression of ESR1.** (A) The relative *MMAA* mRNA expression was promoted in two cell lines as the *ESR1* was overexpressed. (B) The MMAA expression was increased in two cell lines as the ESR1 was overexpressed in protein level. ﻿(﻿*P < 0.05, **P < 0.01; ≥ three biological repeats per group.)

**Supplementary Table 1.** Baseline information for HCC patients from TCGA database.

| **Characteristics** | **Total** | **Female (n=120)** | **Male (n=249)** | **P value** |
| --- | --- | --- | --- | --- |
| **Age** |  |  |  |  |
| NA | 1 | 0 | 1 | 0.2228 |
| < 60 | 169 | 48 | 121 |  |
| ≥ 60 | 199 | 72 | 127 |  |
| **AJCC T stage** |  |  |  |  |
| NA | 2 | 1 | 1 | 0.2623 |
| T1-2 | 275 | 87 | 188 |  |
| T3-4 | 92 | 32 | 60 |  |
| **AJCC stage** |  |  |  |  |
| NA | 24 | 10 | 14 | 0.7442 |
| I-II | 257 | 77 | 180 |  |
| III-IV | 88 | 33 | 55 |  |
| **Grade** |  |  |  |  |
| NA | 5 | 3 | 2 | 0.296 |
| G1-2 | 232 | 71 | 161 |  |
| G3-4 | 132 | 46 | 86 |  |
| **BMI** |  |  |  |  |
| NA | 34 | 14 | 20 | 0.5133 |
| < 25 | 177 | 55 | 122 |  |
| ≥ 25 | 158 | 51 | 107 |  |

**Supplementary Table 2.** The clinicopathological features of 55 HCC patients included from our center.

|  |  | | **MMAA expression** | |  |
| --- | --- | --- | --- | --- | --- |
|  | **Cases** | | **Low %** | **High %** | **P value** |
| **Total** | 55 | | 42 (76.4%) | 13 (23.6%) |  |
| **Age** |  | |  |  |  |
| < 60 |  | | 34 | 10 | 0.751 |
| ≥ 60 |  | | 8 | 3 |  |
| **Sex** |  | |  |  |  |
| Male |  | | 32 | 10 | 0.9567 |
| Female |  | | 10 | 3 |  |
| **AFP (ng/ml)** |  | |  |  |  |
| < 400 |  | | 28 | 7 | 0.4011 |
| ≥ 400 |  | | 14 | 6 |  |
| **HBsAg** |  | |  |  |  |
| Negative |  | | 21 | 8 | 0.4665 |
| Positive |  | | 21 | 5 |  |
| **Size (cm)** |  | |  |  |  |
| < 5 |  | | 20 | 8 | 0.3803 |
| ≥ 5 |  | | 22 | 5 |  |
| **Recurrence** |  | |  |  |  |
| No |  | | 23 | 6 | 0.587 |
| Yes |  | | 19 | 7 |  |
| **Liver cirrhosis** | |  |  |  |  |
| No |  | | 25 | 8 | 0.8969 |
| Yes |  | | 17 | 5 |  |

**Supplementary Table 3.** Detail information for the primers we used in our study. F, forward; R: reverse.

| **Gene names** | **Sequences (5′-3′)** |
| --- | --- |
| MMAA-F | AGAGCACCTTTCCGATGTTACC |
| MMAA-R | AGTCCTTCTGTGTGGTCCTTTA |
| GCLC-F | GTGGTACTGCTCACCAGAGTG |
| GCLC-R | AGCTCCGTGCTGTTCTGGGCCTT |
| GCLM-F | ATCTTGCCTCCTGCTGTGTGATGC |
| GCLM-R | CAATGACCGAATACCGCAGTAGCC |
| ME1-F | CTGCTGACACGGAACCCTC |
| ME1-R | GATCTCCTGACTGTTGAAGGAAG |
| TXNIP-F | GGTCTTTAACGACCCTGAAAAGG |
| TXNIP-R | ACACGAGTAACTTCACACACCT |
| HMOX1-F | AAGACTGCGTTCCTGCTCAAC |
| HMOX1-R | AAAGCCCTACAGCAACTGTCG |
| FOXM1-F | CGTCGGCCACTGATTCTCAAA |
| FOXM1-R | GGCAGGGGATCTCTTAGGTTC |
| NRF2-F | CCAATTCAGCCAGCCCAGCACAT |
| NRF2-R | CAGGTGACTGAGCCTGATTAGTAG |
| ESR1-F | CCCACTCAACAGCGTGTCTC |
| ESR1-R | CGTCGATTATCTGAATTTGGCCT |
| ACTB-F | CATGTACGTTGCTATCCAGGC |
| ACTB-R | CTCCTTAATGTCACGCACGAT |

**Supplementary Table 4.** Detail information for the primers we used in ChIP. F, forward; R: reverse.

| **Fragments** | **Sequences (5′-3′)** |
| --- | --- |
| P1-F | CAAAGGCCAAATTCCTTTCA |
| P1-R | CAGCTGATCAAGGCTGTGTG |
| P2-F | AGACAGTGGGGTCATGGGTA |
| P2-R | TCAGTGGCTCAGTTGCTGAC |
| P3-F | CATGGTCCCCACTTTAGGAA |
| P3-R | GATGCAACAGGAAGGCTCTC |
